# Characterization of Maize Near-Isogenic Lines With Enhanced Flavonoid Expression to Be Used as Tools in Diet-Health Complexity

**DOI:** 10.3389/fpls.2020.619598

**Published:** 2021-01-18

**Authors:** Binning Wu, Haotian Chang, Rich Marini, Surinder Chopra, Lavanya Reddivari

**Affiliations:** ^1^Department of Plant Science, The Pennsylvania State University, State College, PA, United States; ^2^Interdisciplinary Graduate Program in Plant Biology, The Pennsylvania State University, State College, PA, United States; ^3^Department of Food Science, Purdue University, West Lafayette, IN, United States

**Keywords:** maize, near-isogenic line, anthocyanin, flavan-4-ol, phlobaphene, antioxidant capacity

## Abstract

Increasing incidence of chronic diseases in the 21st century has emphasized the importance of developing crops with enhanced nutritional value. Plant-based diets are associated with reduced incidence of many chronic diseases. The growing population and increased food demand have prioritized the development of high-yielding commercial crop varieties at the expense of natural flavors as well as health-benefiting compounds including polyphenols. Flavonoids are a large subfamily of polyphenols abundant in the plant kingdom with known health-promoting effects, making them a promising trait to be re-introduced into elite lines. Given the vast array of flavonoids and the complexity of plant food metabolome interactions, it is difficult to identify with certainty the specific class(es) of flavonoids in the food matrix that are anti-inflammatory. To address this, we have developed four maize near-isogenic lines (NILs); a line that lacked both anthocyanins and phlobaphenes, a second NIL containing phlobaphenes, a third line had anthocyanins, and a fourth line that contained both anthocyanins and phlobaphenes. The phytochemical profiles and the antioxidant potential of the NILs were characterized. The accumulation of anthocyanins and phlobaphenes contributed significantly to antioxidant capacity compared to maize lines that lacked one or both of the compounds (*p* < 0.05). Pilot study showed that intake of flavonoid-rich maize diets were able to alleviate experimental colitis in mice. These NILs offer novel materials combining anthocyanins and phlobaphenes and can be used as powerful tools to investigate the disease-preventive effects of specific flavonoid compound in diet/feeding experiments.

## Introduction

The prevalence of chronic diseases globally is a major challenge in the 21st century. Growing evidence associates plant-based diets with reduced risk of many chronic diseases including cardiovascular disease (CVD), chronic pulmonary disease, chronic kidney disease, diabetes, obesity, hypertension and inflammatory bowel disease. These health beneficial effects of plant-based diets are due in part to the presence of bioactive polyphenols (Temple and Gladwin, 2003; Hever and Cronise, 2017; Zhai et al., 2018; Sarker and Oba, 2019a, c; Joshi et al., 2020).

Flavonoids, a subclass of polyphenols, are being studied extensively to explore their disease-preventing effects and mechanisms of their actions. These compounds are capable of interfering with electron-transfer reaction pathways to scavenge free radicals (Sarker and Oba, 2019b, 2020b; Sarker et al., 2020a), making them promising candidates to counteract inflammation-associated chronic diseases. Though the health-promoting effects of phenolic compounds and flavonoids are well known (Sarker and Oba, 2020c, d, e; Sarker et al., 2020b), clinical studies using purified phytochemicals have yielded conflicting results (Liu, 2004; Vezza et al., 2016). For example, a study of more than 11,000 male physicians taking 50 mg β-carotene supplements daily for over 11 years showed no significant changes in terms of CVD incidence as compared to a placebo-receiving group (Hennekens et al., 1996). β-carotene and vitamin E supplements also failed to lower the incidence of lung cancer among male smokers (The Alpha-Tocopherol, Beta Carotene Cancer Prevention Study Group, 1994). In a study with atrophic gastritis patients, daily consumption of vitamin C for 5 years was not effective in ameliorating high blood pressure (Kim et al., 2002). These observations raise the question do purified phytochemicals confer the same health benefits as they do in their naturally occurring forms?

Studies over the past two decades have demonstrated the connection between structural alterations and bioavailability of phytochemicals and emphasized the importance of the whole-food matrix. An example of this is quercetin. In a dextran sulfate sodium-induced colitis mice model, ingestion of quercetin was devoid of an anti-inflammatory effect, whereas ingestion of its glycoside rutin displayed potent protective effects (Kwon et al., 2005). Matrix-bound phytochemicals exerted higher and prolonged antioxidant activity than their free forms, as the digestion process led to a continuous release of these health beneficial compounds that are bound to cell wall polysaccharides to regulate their bioaccessibility (Rondini et al., 2004; Vitaglione et al., 2008; Cömert and Gökmen, 2017). Though it is suggested to utilize phytochemicals in original forms to retain their efficacy, whole-food studies have the downside of being extremely difficult to pinpoint contributions of any single class of compounds from a complex nutritional background. The development of near-isogenic lines (NILs) differing in specific compounds offers a powerful alternative to elegantly address the above concerns (Martin et al., 2011; Sestari et al., 2014). NILs are generated by a cross of donor parent and recurrent parent, followed by several backcrosses with the recurrent parent to achieve highly homozygous genetic backgrounds that differ only in a single or a few genes (Keurentjes et al., 2007). Having the advantages of being able to minimize background genetic effects, NILs are heavily used in Quantitative Trait Locus (QTL) mapping to locate genes involved in complex quantitative traits such as disease resistance (Yuan et al., 2017).

Maize (*Zea Mays* L.) is one of the most important food crops worldwide and is widely used as food, feed and feedstock for biofuels. The long-lasting trend to breed for high-yield maize cultivars has led to the loss in phytochemicals, as evidenced by the majority of commercial maize lines being colorless in both kernel pericarp and aleurone (Casas et al., 2014). Though there are commercialized colored maize lines, they are appreciated only by a restricted market (Capocchi et al., 2017).

**FIGURE 1:**
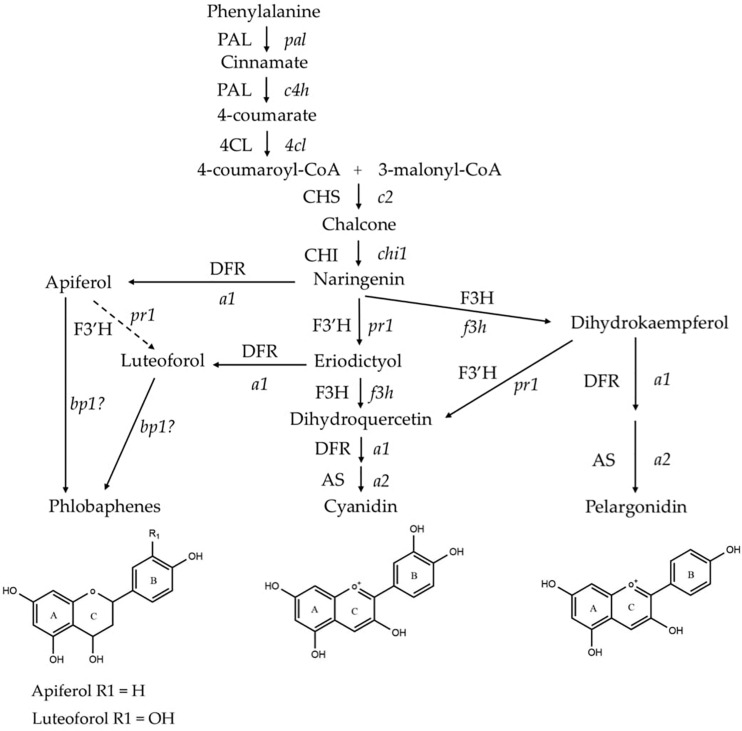
Phenylpropanoid biosynthetic pathway of anthocyanins (cyanidin and pelargonidin) and phlobaphenes in maize. Genes (enzymes) involved in the pathway are: *pal* (PAL), phenylalanine-ammonia lyase; *c4h* (C4H), cinnamic acid hydroxylase; *4cl* (4CL), 4-coumaryl-CoA ligase; *c2* (CHS), chalcone synthase; *chi1* (CHI), chalcone isomerase; *f3h* (F3H), flavanone 3-hydroxylase; *pr1* (F3′H), flavonoid 3′-hydroxylase; *a1* (DFR), dihydroflavonol reductase; *a2* (AS), anthocyanidin synthase; and *brown pericarp1* (*bp1*). (Adapted from Sharma et al., 2011, 2012).

Flavonoid biosynthesis pathway in maize has been intensively studied, among which sub-branches of anthocyanin and phlobaphene biosynthesis pathways are well characterized at genetic and molecular levels. Anthocyanins and phlobaphenes are synthesized through the phenylpropanoid pathway, with naringenin as the pathway deciding point (Figure 1). Flavan-3,4-diols such as dihydroquercetin and dihydrokaempferol are precursors of purple (cyanidin) and red (pelargonidin) anthocyanins, respectively. Apiferol and luteoferol are flavan-4-ols and precursors of reddish brown compound known as phlobaphenes. In general, there are four structural genes (enzymes) shared by both flavan-4-ol and flavan-3,4-diol pathways: *c2* encoded CHS (Chalcone Synthase), *chi1* encoded CHI (Chalcone Isomerase), *a1* encoded DFR (Dihydroflavonol Reductase) and *pr1* encoded F3′H (Flavonoid 3′ Hydroxylase). In pericarp (outer layer of ovary wall) and cob glumes (papery brackets that subtend the kernel), these genes are transcriptionally and independently regulated by an R2R3-MYB transcription factor Pericarp color1 (P1). Interestingly, in flavan-3,4-diol pathway these genes are regulated by pairs of duplicated transcription factors that have tissue specific expression; Red1/Booster1 (R1/B1) are bHLH proteins that interact with Myb proteins Colorless1/Purple plant1 (C1/PL1) (Chandler et al., 1989; Grotewold et al., 1994, 1998; Sharma et al., 2011, 2012). Anthocyanin accumulation requires R1 + C1 for aleurone and B1 + PL1 for plant body (vegetative tissues). Although the Myb domain P1 and C1 are over 70% homologous (Grotewold et al., 1994), only C1, but not P1, interacts with R1 to activate anthocyanin biosynthesis in maize (Grotewold et al., 2000). Anthocyanins act as antioxidants with verified ability to block pro-inflammatory cytokine signaling cascades to alleviate inflammation (Li et al., 2019). Phlobaphenes and anthocyanins serve as potential UV protectants in plants (Rius et al., 2012, 2016; Cassani et al., 2017). Studies have demonstrated that phlobaphene-rich maize lines possess higher antioxidant activity than phlobaphene-lacking lines (Casas et al., 2014; Wu et al., 2020).

Previous studies using isogenic food materials in investigating the health-promoting properties of phytochemicals have verified the feasibility of the NIL strategy and yielded promising results. For example, studies used maize NILs differ in *r1* or *b1*/*pl1* constitutes showed that anthocyanin-rich diet offered cardioprotection (Toufektsian et al., 2008, 2011; Petroni et al., 2017) and neuroprotection (Magni et al., 2018) in rodent models. Tomlinson et al. (2017) used extracts from two tomato NILs with enhanced flavonoids accumulation to treat peptidoglycan-challenged murine colonic epithelial cells. They reported that a 2% extract treatment has significantly downregulated the production of IL-6 and TNF-α, and suppressed the p38 MAPK and STAT3 pathways. Another study found that dietary supplementation using tomato NILs with stacked flavonols, anthocyanins, and stilbenoids was effective in alleviating experimental colitis in mice (Scarano et al., 2018). Despite these scientific records, NIL materials available for assessing other phytochemical classes remain scarce. More importantly, sparsity of NIL with stacked nutrients has limited the investigation of potential synergy of phytochemicals in promoting health.

We hypothesized that NILs that express transcription factors r1 or p1 show accumulation of anthocyanins or phlobaphenes, respectively and these NILs can be used to understand the health benefits of individual classes of compounds with in the food matrix. Accordingly, this study aimed to develop and characterize maize NILs with differential flavonoids accumulation to aid in research on the health-promoting properties of phytochemicals, and to test the prophylactic efficacy of anthocyanins and phlobaphenes in animal diet experiment in a disease model. Four maize NILs that differ only in anthocyanins and phlobaphenes expression in kernels were developed, namely ‘A’ (lacks both anthocyanins and phlobaphenes), ‘B’ (contains phlobaphenes only), ‘C’ (contains anthocyanins only) and ‘D’ (contains both anthocyanins and phlobaphenes), characterized their phytochemical profile via targeted and global metabolomics, and investigated the spatial and temporal accumulation of anthocyanins and phlobaphenes. Compared to a flavonoid-lacking line, flavonoid-rich maize lines possess greatly increased antioxidant activity, a potential health-promoting effect worth exploring further. In addition, the almost identical genetic background makes the four NILs powerful tools to be incorporated into diet experiments to investigate the disease-preventive effect of specific flavonoids. Here we further performed an experimental colitis study in mice which demonstrates the anti-colitis potential of maize anthocyanins and phlobaphenes within a whole-food matrix.

## Materials and Methods

### Chemicals

Six anthocyanin chloride standards (cyanidin, peonidin, malvidin, pelargonidin, petunidin, and delphinidin), pelargonidin-3-glucoside and petunidin-3-glucoside were obtained from Indofine Chemicals (Hillsborough, NY, United States). Seven phenolic acid standards (protocatechuic acid, chlorogenic acid, caffeic acid, ferulic acid, coumaric acid, gallic acid, and sinapic acid), L-methionine sulfone and 2,2-diphenyl-1-picrylhydrazyl were obtained from Sigma-Aldrich (St. Louis, MO, United States). Dextran sulfate sodium (DSS, 4 KDa) salt was obtained from Alfa Aesar (Haverhill, MA, United States). Other chemicals were obtained from Fisher Scientific (Hampton, NH, United States) unless otherwise mentioned.

### Maize Stock and Plant Materials

To develop near-isogenic lines, maize inbred line 4Co63 (referred to as line ‘A’ in this study) was obtained from the National Seed Storage Laboratory (Fort Collins, CO, United States). Line A has no phlobaphenes in pericarp and cob glumes because it carries a *p1-ww* (white pericarp, white cob glume) as well it has no anthocyanins accumulation in aleurones because of *r1*. Genetic stocks *P1-rr-4B2* and *p-del2* were obtained from Dr. Thomas Peterson, Iowa State University, Ames, IA. *P1-rr-4B2* carries a functional *P1-rr* (red pericarp, red cob glume) allele which was derived from *P1-vv* (variegated pericarp, variegated cob glume) by intragenic transposition of a transposon *Activator* (Grotewold et al., 1991). To develop NIL ‘B’, genetic stock *P1-rr-4B2* was used as female crossed with inbred line 4Co63 used a male parent and the F_1_ (female) was backcrossed six times with 4Co63 as a recurrent male parent, while selecting for the *P1-rr* seed phenotype. To develop line ‘C,’ genetic stock *p-del2* (*p1, p2, C1*, and *R1*) (Zhang et al., 2000) was used a female and crossed with 4Co63 and the F_1_ (female) was backcrossed six times with 4Co63 as a recurrent male parent, while selecting for seeds with purple aleurones, colorless pericarp, and colorless cob glumes. Line C thus accumulated anthocyanins in aleurone layer while it had white pericarp and white cob glumes. Line ‘D’ was developed by crossing line ‘B’ as a female and line ‘C’ as a male. The resulting F_1_ (female) was backcrossed five times with line C as a male parent while selecting seeds with purple aleurones, red pericarp and red cob glume in subsequent generations. Pericarp is a maternal tissue, while aleurone shows a Xinia effect and thus to develop NILs B, C, and D, all F_1_s were used as females and recurrent parents as males to ensure full expression of pericarp and aleurone flavonoids in the backcrossed ears. Reciprocal crosses were not performed in order to ensure selection of pericarp phenotypes in the F_1_ generation for lines B and D. All four NILs were planted and self-pollinated in the summer of 2018 at the Penn State Russel Larson Agronomy Research farm, Rocksprings, PA, United States. Ears were harvested and kernels were collected at 10, 14, 18, 24, and 45 days after pollination (DAP). One set of kernels was immediately stored at −80°C whereas another set was further dissected into pericarp, endosperm with aleurone, and embryo before storing at −80°C for tissue-specific biochemical analyses.

### Gene Expression Assay

Total maize kernel RNA was extracted with PureLink RNA Mini Kit (Thermo Fisher Scientific, Waltham, MA, United States). The concentration of the isolated RNA was determined by Take3 plate of Cytation3 microplate reader. Reverse transcription of 1 μg RNA was performed using SuperScript IV VILO Master Mix (Invitrogen, Carlsbad, CA, United States) following manufacturer’s protocol. Quantitative real-time PCR (qRT-PCR) was performed using PerfeCTa SYBR Green FastMix (Quantabio, Beverly, MA, United States) and the following gene-specific primers (Integrated DNA Technologies, Coralville, IA, United States): C1 forward: 5′-TCGGACGACTGCAGCTCGGC-3′; C1 reverse: 5′-CACCGTGCCTAATTTCCTGTCCGA-3′; R1 forward: 5′-ATGGCTTCATGGGGCTTAGATAC-3′; R1 reverse: 5′-GAATGCAACCAAACACCTTATGCC-3′ (Sharma et al., 2011); P1 forward: 5′-TCCGGTGCGGCAAGAG-3′; P1 reverse: 5′-GGAGCTTGATGATGATGTCTTCTTC-3′. The program consisted of: hold stage at 95°C for 10 min, followed by 45 cycles of PCR stage, 15 s denaturation at 95°C, 1 min annealing at 60°C and 30 s extension at 72°C. Results were normalized to endogenous control maize β-Actin (forward: 5′-CCTTGGAATGCCCAGCAATG-3′; reverse: 5′-GAGGATCTTCATTAGGTGGT-3′) and expressed as target gene to β-Actin ratio. Total RNA was isolated from mice distal colonic tissue preserved in RNAlater (Thermo Fisher, Waltham, MA, United States) using PureLink RNA Mini Kit (Invitrogen, Carlsbad, CA, United States) according to the manufacturer’s instructions and was reverse transcribed using SuperScript IV VILO Mater Mix (Thermo Fisher). The expression levels of Interleukin-6 (IL-6) were quantified by quantitative real-time PCR (qRT-PCR) using pre-designed duplex Taqman gene expression assays (Thermo Fisher; Mm01210733_ml). Results were normalized to internal control β-Actin (Mm02619580_gl).

### Tissue Sample Extraction for Metabolites

Sample extraction was performed by Bligh–Dyer method using methanol containing 0.1% formic acid, water containing 0.1% formic and chloroform (Bligh and Dyer, 1959). Briefly, 600 μL acidified methanol was added to 50 mg maize sample followed by vigorous vortex and sonication, then 540 μL acidified water and 600 μL chloroform were added. Samples were shaken for 2 min and centrifuged at 4°C, 5,000 × *g* for 30 min. The aqueous phase was transferred into a new microcentrifuge tube and stored at −20°C for targeted and global metabolomics, total phenolics assay and antioxidant activity assay.

### Targeted Metabolomics

The aqueous phase from above was evaporated to dryness in a vacuum concentrator (Eppendorf, Hauppauge, NY, United States). The dried fraction was reconstituted in 75 μL 80% methanol containing 0.1% formic acid and 20 μM L-Methionine sulfone as an internal standard (IS), followed by 2 min vortex and 8 min centrifugation at 16,000 × *g*. The reconstituted samples were used for downstream analysis. For a standard curve, standard mixtures containing 17 target compounds were prepared at the following five concentrations: 3,330, 1,665, 333, 33.3, and 3.33 ng/mL via serial dilution using the same solvent as used for sample preparation. An Agilent 6460 QQQ coupled to an Agilent 1200 Rapid Res LC system was used for the analysis (Palo Alto, CA, United States). LC separation was done by using a Water’s Crop Xbridge reversed-phase C18 2.1 mm × 100 mm, 3.5 μm column (Milford, MA, United States) with a flow rate of 0.35 mL/min. The sample (5 μL) was injected and eluted with a mixture of phase A (5% acetonitrile, 95% ddH_2_O with 0.2% formic acid) and phase B (100% acetonitrile with 0.2% formic acid). The mobile phase gradients were: 0–1 min, 100% A; 1–7 min, from 0% B to 95% B; 7–10 min, isocratic of 95% B; 10–10.10 min, from 95% B to 0% B; 10.10–14 min, 100% A. Electrospray (ESI) interface operated in both positive (for anthocyanin) and negative modes (for phenolic acid) were used for compound quantification (Supplementary Table 1). Data were acquired in multiple reaction monitoring mode (MRM). The source parameters for MS were set as follows: gas temperature 325°C, drying gas flow rate 8.0 L/min, nebulizer pressure 45 psi, sheath gas temperature 250°C, sheath gas flow 7.0 L/min, capillary 3800 V, ΔEMV of + 300 V, nozzle 1000 V(+), and 500 V(−). Data analysis was performed by Agilent MassHunter Quantitative analysis software (v6.0). Peak area was normalized to IS and concentration was calculated based on a pre-constructed standard curve. Anthocyanins and phenolic acids content were expressed as microgram per 100-g sample dry weight.

### Global Metabolite Profiling

The upper aqueous phase was evaporated to dryness in a vacuum concentrator. The dried fraction was reconstituted in 50 μL diluent that composed of 95% water and 5% acetonitrile containing 0.1% formic acid, followed by 2 min vortex and 8 min centrifugation at 16,000 × *g*. HPLC was performed via an Agilent 1290 system (Palo Alto, CA, United States) using a Waters HSS T3 column (1.8 μm, 2.1 mm × 100 mm) with a flow rate of 0.45 mL/min. The sample (10 μL) was injected and eluted with a mixture of phase A (100% ddH_2_O with 0.1% formic acid) and phase B (100% acetonitrile with 0.1% formic acid). The mobile phase gradients were: 0–1 min, 100% A; 1–16 min, from 0% B to 80% B; 16–22.5 min, from 80% B to 95% B; 22.5–23.5, from 95% B to 0% B; 23.5–28.5 min, hold at 0% B, 100% A. The mass analysis was obtained using an Agilent 6545 Q-TOF mass spectrometer with ESI capillary voltage +3.5 kV, nitrogen gas temperature 325°C, drying gas flow rate 8.0 L/min, nebulizer gas pressure 30 psi, fragmentor voltage 130 V, skimmer 45 V, and OCT RF 750 V. Mass data (from m/z 70–1000) were collected at 3 spectra/s using Agilent MassHunter acquisition software (v.B.06). Mass accuracy was improved by infusing Agilent Reference Mass Correction Solution (G1969-85001). Peak deconvolution, integration, and alignment were performed using Agilent ProFinder (v.B.08). Bioinformatics and principal coordinate analysis (PCA) was performed using MetaboAnalyst 4.0 (McGill University, Montreal, QC, Canada). Peak areas were normalized by log transformation. Significance analysis was performed by one-way ANOVA with Tukey *post hoc* and two-way ANOVA with a false discovery rate (FDR). Metabolites with *p* < 0.001 and fold change > 2 were considered significant. Peak annotations were performed using the Plant Metabolic Network^1^ CornCyc9.0 database with a mass error of less than 30 ppm.

### Maize Flavan-4-Ols Quantification

Flavan-4-ols quantification was performed as described previously (Grotewold et al., 1998). Briefly, 100 mg maize tissue sample was placed in 1 mL acid butanol (HCl:butanol = 3:7, v/v) and incubated for 1 h at 37°C, followed by 20 s centrifugation at 10,000 × *g*. The supernatant was pipetted into a 96-well plate and sample absorbance spectrum from 250 to 600 nm was obtained using Cytation3 microplate reader (BioTek, Winooski, VT, United States), flavan-4-ols content is reported as absorbance at 565 nm per gram sample used. To verify the existence of flavan-4-ols, supernatant was boiled at 90°C for 10 min and absorbance was measured again with the same instrument settings.

### Total Monomeric Anthocyanin Determination

Total monomeric anthocyanin was determined using the pH-differential method (Lee et al., 2005). Briefly, 20 μl sample was added to 280 μL 0.025 M potassium chloride buffer (pH = 1.0) and sodium acetate buffer (pH = 4.5), respectively and hold for 15 min. Diluent absorbance (A) was recorded at 525 and 700 nm and calculated using the following equation: *A* = (*A*_525_ − *A*_700_)_*pH*__1_._0_ − (*A*_525_ − *A*_700_)_*pH*__4_._5_; Monomeric anthocyanin pigment concentration (MAC) was calculated using the following formula: MAC = (A × 449.2 × dilution factor × 1000)/26,900 × 1. Total monomeric anthocyanin content is expressed as milligram cyanidin-3-glucoside (C3G) equivalents per 100 g sample used.

### Total Phenolic Content Determination

Total phenolic content was determined by Folin–ciocalteu (FC) method described previously (Madiwale et al., 2012). Gallic acid (GA) solutions at different concentrations were used to construct a standard curve. Briefly, 150 μL 0.2 M FC reagent was added to 35 μL sample and held for 5 min, then 115 μL of 7.5% (w/v) sodium carbonate was added, followed by 30 min incubation at 45°C. Sample absorbance was recorded at 765 nm by Cytation3 microplate reader. Total phenolics content is expressed as milligram of GA equivalents per 100-g sample used.

### Total Antioxidant Activity Determination

Total antioxidant activity was determined by 2,2-diphenyl-1-picrylhydrazyl (DPPH) method (Sarker and Oba, 2020a). Trolox solutions at different concentrations were used to construct a standard curve. Briefly, 285 μL of DPPH solution was added to 15 μL of sample and held for 2 h. Sample absorbance was recorded at 515 nm. Total antioxidant activity is expressed as milligrams of Trolox equivalents per 100-g sample used.

### Mice Health Study

7% corn oil diet (control) (TD.95092), 25% maize supplemented diets line A (TD.190202), line B (TD.190203), line C (TD.190204) and line D (TD.190205) were from Envigo Teklad (Madison, WI, United States). All experimental diets were balanced with respect to calorie intake and macro and micro nutrient composition but differed only in NIL supplementation (Supplementary Table 2). Wild-type male C57BL/6 mice (*n* = 36; 4 weeks old) were purchased from Jackson Laboratory (Bar Harbor, ME, United States) and housed at Purdue University under institutionally approved protocols. Sample size was determined based on our previous data to ensure a >90% statistical power of downstream analysis. Statistical power was calculated using an online tool^2^. All procedures performed were adhered to the Guide for the Care and Use of Laboratory Animals published by the National Institutes of Health. Mice were randomly assigned to six treatment groups (control, DSS, A, B, C, and D) for a total of 9 weeks, both control and DSS group mice were fed on control diet. At the beginning of the ninth week, 3% DSS (w/v) was added to the drinking water for all the treatment groups to induce colitis except for control group. Mice assigned to control group received normal drinking water throughout the experiment. On average, mice consuming C and D diet received around 4.2 and 0.63 mg anthocyanins per kg body weight per day, respectively. Daily intake of phlobaphenes were not quantified because of the difficulty in extracting them in the native form and the lack of standards for precursors apiferol and luteoferol. All mice were sacrificed at the end of the ninth week, mice colons were harvested and measured for colon length. After measurement distal colons were stored in 10% formalin (v/v) for histopathological evaluation. Distal colon tissue sections were stained with hematoxylin and eosin (H&E) and examined by a board-certified veterinary pathologist who was blinded to all the treatment groups. A semi-quantitative method consisting of six parameters was employed to generate colon histology score. To grade mucosal hyperplasia, mononuclear infiltrate and polymorphonuclear leukocyte infiltrate, the following histomorphological scale was used: 3 = marked, 2 = moderate, 1 = mild and 0 = normal. To grade crypt architectural distortion and submucosa involvement, the following histomorphological scale was used: 3 = 50% or greater, 2 = 10% - 50%, 1 ≤ 10% and 0 = normal. To grade epithelial cell death, the following histomorphological scale was used: 3 = ulcerations, 2 = erosions, 1 = superficial epithelial sloughing/single cell necrosis, 0 = normal. Results are presented as cumulative scores.

### Statistical Analysis

All statistical tests were carried out with GraphPad Prism8 (La Jolla, CA, United States) and MetaboAnalyst 4.0. Statistically significant differences were determined using one-way ANOVA with Tukey *post hoc* test (*p* < 0.05), or two-way ANOVA with Tukey *post hoc* test (*p* < 0.05) or false discovery rate (FDR, *p* < 0.01). Values assigned to different letters are significantly different, figure without letter assignment indicates no significant differences between the mean values were detected.

## Results

### Altered Introgression of Transcription Factors Cause Phenotypic and Metabolomic Variation

**FIGURE 2:**
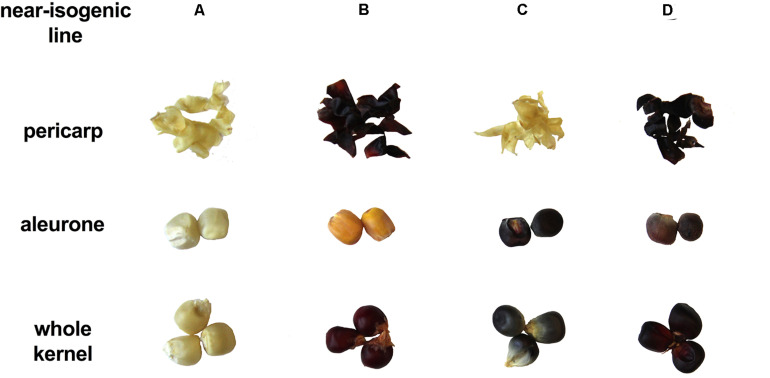
The seed tissue phenotypes of maize NILs used in this study. Picture shows pigmentation of whole kernel, dissected pericarp and endosperm with aleurone layer.

Presence/absence of transcription factor P1, C1, and R1 led to marked pigmentation differences in maize kernels phenotypically (Figure 2). Line ‘A’ is colorless in both pericarp and aleurone layer, suggesting a complete lack of anthocyanins and phlobaphenes. Line ‘B’ had an accumulation of red pigment in pericarp, suggesting the presence of phlobaphenes. Line ‘C’ had a colorless pericarp and purple-colored aleurone layer, suggesting the presence of anthocyanins and the absence of phlobaphenes. Line ‘D’ had the darkest kernel pigmentation out of all the four NILs, because of red-colored pericarp (phlobaphenes) and purple-colored aleurone (anthocyanins).

**FIGURE 3:**
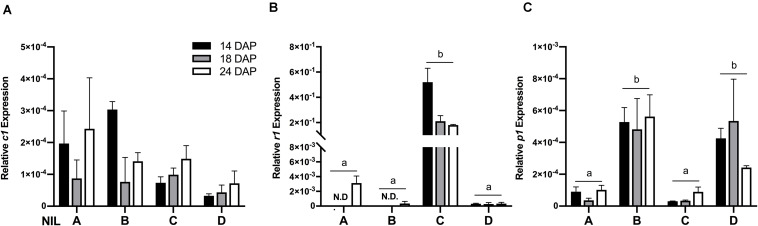
The relative mRNA expression level of *c1* **(A)**, *r1* **(B)**, and *p1* **(C)** in four maize NILs at different developmental stages. Mean values ± s.e.m. of three biological replicates. Two-way ANOVA followed by Tukey’s post test (*p* < 0.05), multiple comparisons were performed by comparing means between genotypes.

To verify whether the observed phenotypic differences were due to varying expression levels of the regulating transcription factors, gene expression assays were performed on *c1*, *r1*, and *p1* by qRT-PCR. Results showed that the expression levels of *c1* were similar across all the four NILs (Figure 3A) and *r1* in lines ‘A’ and ‘B’ had almost non-detectable expression (Figure 3B). Notably, the expression level of *r1* was remarkably higher in line ‘C’ than in ‘D.’ On the other hand, as expected, the expression level of *p1* was significantly higher in line ‘B’ and ‘D’ than in ‘A’ and ‘C’ (Figure 3C). No obvious gene expression pattern was observed across time, indicating the activation of *c1, r1*, and *p1* started at the early grain filling stage.

### Global Metabolite Accumulation Patterns Match Gene Expression Results in the NILs

**FIGURE 4:**
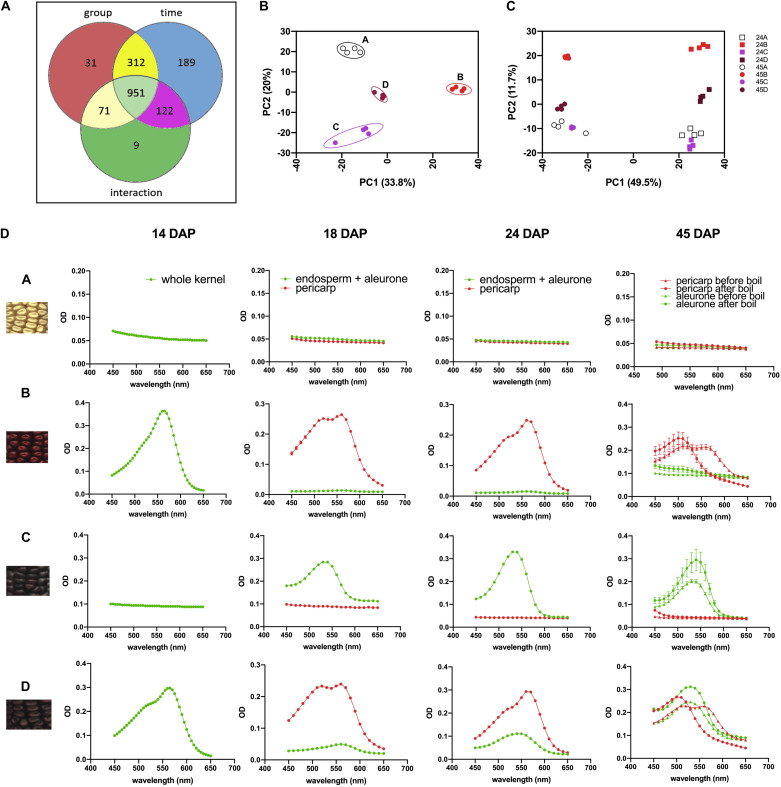
**(A)** Venn diagram illustrating the number of compounds with significantly different abundance affected by group (line A, B, C, D), time (24 and 45 DAP) or the interaction of group and time; **(B)** PCA of the Euclidean distance matrix of 45 DAP NILs metabolites; **(C)** PCA of the Euclidean distance matrix of 24 and 45 DAP NILs metabolites. **(D)** 450–650 nm absorption spectra showing earlier accumulation of phlobaphenes (maximum absorbance around 565 nm) and later accumulation of anthocyanins (maximum absorbance around 540 nm) in NIL B, C and D.

We then investigated the overall metabolomics profiles of the four NILs using global metabolomics. For phlobaphenes detection and quantification we extracted the precursor flavan-4-ols. Two methods were used to extract maize anthocyanins and flavan-4-ols. Anthocyanins are water-soluble and can be easily extracted using methanol solvent whereas flavan-4-ols were extracted with acid butanol (HCl:butanol = 3:7, v/v). In our study, Bligh–Dyer method was used to extract 24 and 45 DAP kernel metabolites for targeted and non-targeted metabolomics, the upper phase extracts (methanol:H_2_O = 2:1.8, v/v) were used for the assay. Peak alignment resulted in 1,685 peaks with significantly different abundance at 24 and 45 DAP (*p* < 0.01, Figure 4A). The majority of the differentially accumulated compounds identified via database were related to phenylpropanoid pathway (Supplementary Table 3). PCA plot showed that the overall metabolite profile of line ‘D’ had shared features with both lines ‘B’ and ‘C’ (Figure 4B). This result matches with the fact that ‘D’ carries functional P1, C1, and R1 allele and accumulates both anthocyanins and phlobaphenes. The involvement of the time factor has caused a distinct separation of maize metabolite profile at 24 and 45 DAP, indicating that time has a greater impact on the overall metabolic profile compared to genotype (Figure 4C).

To investigate the accumulation pattern of anthocyanins and phlobaphenes in the kernels of four NILs, acid butanol extraction and boiling methods were used. Flavan-4-ols are heat-labile and highly unstable under acidic conditions (Stich and Forkmann, 1988). In acidic butanol, both apiferol and luteoferol were converted into their corresponding 3-deoxyanthocyanidins, apigeninidin and luteolinidin, respectively and were visible as cherry red pigments with absorption of λ_*max*_ around 560–565 nm (Stich and Forkmann, 1988; Sharma et al., 2012). This cherry red pigment turns brown after boiling with a λ_*max*_ shifted to 495–500 nm. On the other hand, flavan-3,4-diols are heat stable and have similar λ_*max*_ around 540–545 nm before and after boil. Since anthocyanins accumulate in kernel aleurone whereas phlobaphenes accumulate in kernel pericarp, all the kernels used in the assay were manually dissected except for 14 DAP kernels due to dissection difficulty. Absorbance spectra showed traces of phlobaphenes accumulation in line ‘B’ and ‘D’ as early as 14 DAP, all lines showed no sign of the presence of anthocyanins at 14 DAP (Figure 4D). Absorption peaks of anthocyanins started to show up at 18 DAP in line ‘C’ and ‘D,’ no detection of anthocyanins were observed in ‘A’ and ‘B.’ Different tissues from 45 DAP kernel were used for the boiling test, line ‘A’ showed no absorption peak for both pericarp and aleurone; aleurone of ‘B’ showed no absorption peak whereas pericarp of ‘B’ showed a peak at 565 nm before boiling and shifted to the left after boiling; pericarp of ‘C’ showed no absorption peak whereas aleurone showed a peak at 545 nm and remained similar before and after boiling; pericarp and aleurone of ‘D’ showed a 565 and 545 nm peak, respectively, before boiling, the 565 nm peak shifted to the left whereas the 545 nm peak stayed after boiling. Boiling confirmed the presence of phlobaphenes in NIL B and D, as evidenced by the absorption peak at 565 nm shifted to the left after boiling.

### Flavan-4-Ols and Anthocyanins Accumulation Differ Among Maize Near-Isogenic Lines

Flavan-4-ols accumulation in line ‘B’ and ‘D’ started around 10 DAP, went through a rapid increase from 10 to 14 DAP and stabilized at 14 DAP. From 24 to 45 DAP flavan-4-ols were gradually polymerized into condensed phlobaphenes, showing as increased red kernel pigmentation at 45 DAP. Overall, line ‘D’ had significantly higher flavan-4-ols accumulation than line ‘B’ (Table 1). To chemically characterize major phytochemicals in the four NILs, Bligh-Dyer extracts of 45 DAP kernel were used for targeted metabolomics. Major anthocyanins detected in line ‘C’ and ‘D’ were cyanidin, pelargonidin and petunidin, with cyanidin being the most predominant anthocyanidin (Table 2). Cyanidin content in line ‘C’ was 12 times higher than ‘D,’ whereas pelargonidin content in line ‘D’ was 64 times higher than ‘C,’ suggesting different metabolic shunting patterns within the anthocyanin biosynthesis pathways in line ‘C’ and ‘D.’

**TABLE 1:** Quantification of flavan-4-ols in four NILs at different developmental stages.

Flavan-4-ols (Abs/g kernel)					
Maize NIL	10 DAP^1^	14 DAP^1^	18 DAP^1^	24 DAP^1^	45 DAP^2^
A	0.04	0.11 ± 0.05^*a*^	0.31 ± 0.02^*a*^	0.47 ± 0.04^*a*^	0.40 ± 0.01^*a*^
B	3.77 ± 1.51	12.72 ± 0.74^*b*^	11.00 ± 1.82^*a*^	12.28 ± 4.18^*ab*^	2.98 ± 0.14^*b*^
C	0.06 ± 0.01	0.07 ± 0.01^*a*^	0.40 ± 0.04^*a*^	1.67 ± 0.50^*a*^	0.41 ± 0.02^*a*^
D	0.24 ± 0.03	25.61 ± 2.00^*c*^	23.99 ± 4.52^*b*^	25.67 ± 4.41^*b*^	5.28 ± 0.35^*c*^

**Total anthocyanins (mg C3G equivalent/100 g)^3^**					

**Maize NIL**	**18 DAP**		**24 DAP**	**45 DAP**	

C	0.70 ± 0.26		4.65 ± 1.27	12.3 ± 0.87	
D	0.48 ± 0.15		0.58 ± 0.20	1.80 ± 0.13	

*^1^Fresh kernels were used for the assay; ^2^dry kernels were used for the assay; ^3^Values determined by pH differential method. One-way ANOVA followed by Tukey’s post test, p < 0.05. Mean values ± s.e.m. of three biological replicates. Means within columns followed by common letters do not differ at the 5% level.*

**TABLE 2:** Quantification of major anthocyanins and phenolic acids in four NILs at 45 DAP^1^.

Maize NIL	A	B	C	D
**Anthocyanins (μg/100 g dw)**				
Cyanidin	–	–	7407.92	593.98*
Pelargonidin	–	–	2.34	150.34*
Petunidin	–	–	49.71	115.22*
**Phenolic acids (μg/100 g dw)**				
Protocatechuic acid	3.00^*a*^	4.30^*a*^	28.91^*b*^	14.78^*c*^
*p*-coumaric acid	0.87	2.09	1.46	1.16
Gallic acid	1.57	0.85	0.99	0.89
Caffeic acid	31.27^*a*^	0.02^*b*^	9.66^*c*^	11.51^*c*^
*Trans*-ferulic acid	2.12	2.34	3.03	3.84
Chlorogenic acid	0.59	–	0.09	–

*^1^Results are presented as mean values of three biological replicates. –, below detection limits. Asterisks indicate that the mean values of line ‘C’ and ‘D’ are significantly different, two-tailed Student’s t-test, p < 0.05. Means within rows followed by common letters do not differ at the 5% level, one-way ANOVA followed by Tukey’s post test, p < 0.05. MS parameters are included in Supplementary Table 1.*

### Anthocyanins and Phlobaphenes Contribute to High Phenolic Content and *in vitro* Antioxidant Activity

**FIGURE 5:**
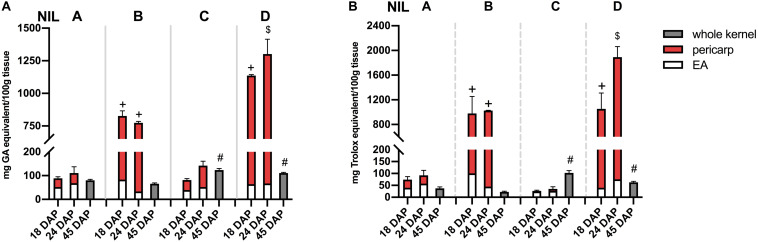
**(A)** Total phenolics content assayed with FC reagent. **(B)** Total antioxidant activity assayed with DPPH. EA, endosperm with aleurone layer; +, significantly higher than NIL A and C; $, significantly higher than NIL A, B, and C; #, significantly higher than NIL A and B whole kernel. Mean values ± s.e.m. of three biological replicates. One-way ANOVA followed by Tukey’s post test, *p* < 0.05.

Phlobaphene-containing pericarp in line ‘B’ and ‘D’ considerably contributed to total phenolics content and conferred significantly higher antioxidant activity compared to line ‘A’ and ‘C’ (Figures 5A,B). At 45 DAP, line ‘C’ had higher phenolics content and antioxidant activity due to increased anthocyanin accumulation. Accumulation of anthocyanins also conferred ‘C’ with significantly higher antioxidant activity compared to ‘A.’ Polymerized flavan-4-ols from 45 DAP dried kernels could not be efficiently extracted with methanol, which led to a lower total phenolics content and antioxidant activity in line ‘B’ and ‘D’ compared to ‘C.’

### Mice Consuming NIL-Supplemented Diets Indicate Anti-colitic Potentials of Maize Anthocyanins and Phlobaphenes

**FIGURE 6:**
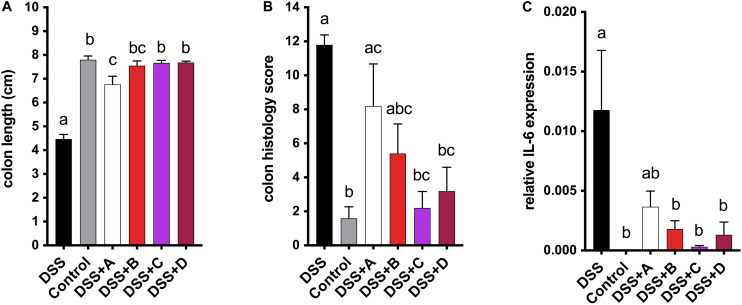
**(A)** Endpoint mice colon length; **(B)** cumulative distal colon histopathology scores; **(C)** relative mRNA expression level of inflammatory marker IL-6. Mean values ± s.e.m. of six biological replicates. One-way ANOVA followed by Tukey’s post test, *p* < 0.05.

To investigate the health-promoting effects of maize anthocyanins and phlobaphenes in a whole-food matrix, we formulated four diets supplemented with 25% of corn meal from each line ‘A,’ ‘B,’ ‘C,’ and ‘D’ as feeding materials and conducted a pilot study on mice. DSS was added to the drinking water to induce acute colitis in mice, the reduction of colon length is an indicator of inflammation onset (Diaz-Granados et al., 2000). After 1 week of DSS exposure, mice fed on B, C, and D diet were protected from colitis-associated colon shortening and mucosal and submucosal damage as compared to the DSS group (Figures 6A,B and Supplementary Figure 1). Moderate colon length restoration was also observed in mice fed on A diet, however, the protective effect was less effective in comparison with that of C and D diets. Mice consuming flavonoid-rich diets also had attenuated gene expression levels of pro-inflammatory cytokine IL-6 (Figure 6C). These results indicate the anti-colitic potentials of matrix-bound anthocyanins and phlobaphenes.

## Discussion

Flavonoid pigmentation in maize has a longstanding interest in genetic studies due to its trait simplicity and phenotypic visibility. In maize, the regulatory role of *p1* in flavonoid biosynthesis pathway has been studied intensively for over 25 years. In maize, *p1* is primarily expressed in pericarps, cob glumes, tassel glumes, silks, and husks and transcriptionally regulates a subset of genes required for flavones and phlobaphene biosynthesis (Grotewold et al., 1991, 1994). On the other hand, the direct interaction of C1 Myb and bHLH protein R1 is required for transcriptional regulation of genes required for anthocyanin biosynthesis in maize aleurones (Grotewold et al., 2000).

The development of maize NILs with different pigmentation and allelic patterns such as *p1-ww* (white pericarp, white cob glumes), *P1-wr* (white pericarp, red cob glumes) and *P1-rr* (red pericarp, red cob glumes) has facilitated studies on *p1*. The role of P1 as a broad-spectrum regulator has been supported by a study where comparative transcriptome analyses were performed on NILs carrying *p1-ww* and *P1-rr*. The RNAseq data revealed a total of 5,565 differentially expressed genes, most of which were associated with phenylpropanoid-lignin or flavonoid biosynthesis pathway (Morohashi et al., 2012). Our global metabolomics data seems to follow a similar trend, the majority of compounds identified with significantly different areas under the curve were involved in the phenylpropanoid pathway. Variations in the metabolic profile of the four NILs assayed in this study could be attributed to varied transcriptional and post-transcriptional regulation of *p1*. This may also explain the higher flavan-4-ols content in line ‘D’ than in ‘B’ despite the similar genetic background. Genetic and epigenetic variation at *p1* alleles (Chopra et al., 1996) has further allowed identification of modifiers such as *unstable factor for orange1* (*ufo1*) that can induce epigenetic gene silencing (Chopra et al., 2003; Wittmeyer et al., 2018). Interestingly, the epigenetic regulation of *p1* is also affected by levels of UV-B radiations. DNA methylation at *p1* was inversely associated with UV-B exposure (Rius et al., 2012, 2016). Our four NILs can be a useful tool for the identification of additional modifiers that may selectively affect accumulation of different flavonoids.

Major anthocyanins detected in line ‘C’ and ‘D’ were cyanidin and pelargonidin, this result lines up with other reports that characterize anthocyanin from colored maize kernels (Salinas Moreno et al., 2005; Camelo-Méndez et al., 2016). Interestingly, we also noticed that cyanidin content in NIL ‘C’ was 12 times higher than that in ‘D,’ whereas pelargonidin content in ‘D’ was 64 times higher than that in ‘C.’ This could be a consequence of resource allocation due to exceeding demand for F3′H (Figure 1). In maize aleurone cells, *pr1* allele producing a non-functional *f3*′*h* results in the accumulation of pelargonidin instead of cyanidin in *Pr1* (Sharma et al., 2011). However, F3′H also is subjected to P1 regulation and is required for phlobaphene biosynthesis to catalyze the conversion of apiferol into luteoferol (Sharma et al., 2012). With both phlobaphene and anthocyanin pathways activated in line ‘D,’ insufficient F3’H could cause less conversion of dihydrokaempferol to dihydroquercetin, resulting in higher pelargonidin accumulation and pigmentation variation between line ‘C’ and ‘D.’ Studies also showed that *r1* is subjected to epigenetic regulations, which lead to paramutation in maize. In the crossing experiment, the weakly expressed *r1*′ allele could transfer the strongly expressed *r1* allele into *r1*′ in a reversible and heritable manner (Brink, 1956). The lower *r1* expression level in line ‘D’ is intriguing and could explain the overall less anthocyanin accumulation in ‘D’ compared to ‘C.’ Interaction between C1 and R1 are highly specific. The first helix of R3 Myb repeat of C1, as well as the last 12 amino acids of the R2 motif, were shown to be required for the binding of C1 to R1. This interaction activates the transcription of *Bz1*, a gene encodes for UDP-glucose flavonoid 3-*O*-glucosyltransferase (UFGT) essential for anthocyanin biosynthesis in maize (Grotewold et al., 2000).

The accumulation of phlobaphene in line ‘B’ and ‘D’ were found to correspond to a high mRNA expression level of *p1*, though no obvious expression patterns were observed. Our result is consistent with Sharma et al. (2012) where the authors reported a higher binding frequency of P1 and *pr1* promoter in *P1-rr* than in *P1-ww* at 15 DAP. Total phenolics assay and DPPH assay results revealed phlobaphenes in the kernel pericarp as dominant contributors, however, due to extraction difficulties of phlobaphenes from kernels at 45 DAP, this pattern was not observed at this stage. The great antioxidant capacity of phlobaphene was also verified and reported by other researchers, making it a desirable trait to be introduced into elite cultivars for a better dietary benefit (Casas et al., 2014; Capocchi et al., 2017; Wu et al., 2020).

Despite the great antioxidant capacities of flavan-4-ols and phlobaphenes, their health-promoting effects, especially from a whole-food background, have not yet been fully investigated and understood. Anthocyanins, conversely, have been studied for their health-promoting effects in various models (He and Giusti, 2010; Li et al., 2019). The development of novel therapeutic agents as a part of future treatments for chronic diseases requires dissected health-benefits conferred by individual phytochemicals to be carefully investigated. However, studies using whole-food or plant extracts have the downside of pinpointing the contribution of any single component from a mixture of nutrients. Moreover, the typically used purified compounds not tethering to the food matrix may have altered chemical forms and varied bioavailability to confound precise dietary suggestions (Martin et al., 2011; Cassidy and Minihane, 2017; Cömert and Gökmen, 2017). The development of NILs opens up new opportunities to address these concerns by proving phytochemicals in their original forms with high genetic homozygosity to minimize background variations. Despite very few scientific attempts, previous studies have demonstrated the feasibility and effectiveness of using NILs in diet experiments to investigate the health benefits of a few specific phytochemicals. One study used two tomato NILs with contrasting anthocyanin accumulation to feed cancer-prone *Trp53*^–/–^ mice has yielded positive results. This study reported that mice fed anthocyanin-rich tomato diet had significantly prolonged lifespan compared to those fed anthocyanin-free tomato diet (Butelli et al., 2008). Scarano et al. (2018) reported the anti-colitis effect of anthocyanins in IBD mice model examined by four tomato NILs. Under 1% DSS exposure, mice consumed anthocyanin-rich tomato diets showed a significantly diminished IL-6 and TNF-α protein production (Scarano et al., 2018). The cytokine inhibitory effects of tomato anthocyanins were also confirmed in a cell-based model (Tomlinson et al., 2017). A previous study used two maize NILs, flavan-4-ols-rich and flavan-4-ols-free maize, as diet supplements in an experimental colitis model in mice. Results showed that a flavan-4-ols enriched maize diet was able to alleviate low-grade colonic inflammation by restoring intestinal barrier function (Wu et al., 2020). The prophylactic efficacy of specific flavonoids were further investigated in a pilot mice study, where anthocyanins and phlobaphenes demonstrated anti-colitis potential. In accordance with recent studies, we found that maize flavonoids offered protection from DSS-induced intestinal epithelial damage and downregulated the expression of pro-inflammatory mediator IL-6.

In conclusion, the flavonoid-rich NILs possess significantly higher phenolics content and antioxidant activity compared to the null line. Combining the health benefits of colored maize lines observed in the pilot study and reported by other studies (Lao et al., 2017; Xiong et al., 2019), maize NILs developed from this study are powerful tools and could be of great help to investigate the potential health-promoting effects of anthocyanins and phlobaphenes.

## Data Availability Statement

The original contributions generated for this study are included in the article/Supplementary Material, further inquiries can be directed to the corresponding author/s.

## Ethics Statement

The animal study was reviewed and approved by The Purdue Institutional Animal Care and Use Committee.

## Author Contributions

SC and LR: conceptualization, resources, and funding acquisition. LR: methodology. BW and HC: validation and investigation. BW: formal analysis and writing—original draft preparation. RM, SC, and LR: writing—review and editing. All authors contributed to the article and approved the submitted version.

## Conflict of Interest

The authors declare that the research was conducted in the absence of any commercial or financial relationships that could be construed as a potential conflict of interest.

## Footnotes

1 plantcyc.org

2 https://www.sphanalytics.com/statistical-power-calculator-using-average-values/
