# Supplementary material for: Characterization of Maize Near-Isogenic Lines With Enhanced Flavonoid Expression to Be Used as Tools in Diet-Health Complexity
Source: Front Plant Sci. 2021 Jan 18;11:619598. doi: 10.3389/fpls.2020.619598 (PMC7874058; doi:10.3389/fpls.2020.619598)
Supplement: Supplementary file 1 [file Data_Sheet_1.DOCX]

Supplementary Material





**Supplementary Figure 1.** (A) Mice body weight loss after DSS exposure. (B) Representative images of mice distal colonic tissue longitudinal section stained with H&E. Data are expressed as means ± s.e.m (n = 6 per group). Significance level (*p* < 0.05) was determined by one-way ANOVA followed by Tukey’s post-test. Colored asterisks indicate the mean value of the corresponding treatment group was significantly different from that of DSS group.
